# Mortality in patients with COVID-19 versus non-COVID-19- related acute respiratory distress syndrome: A single center retrospective observational cohort study

**DOI:** 10.1371/journal.pone.0286564

**Published:** 2023-06-02

**Authors:** Yu-Hsiang Hsieh, Hou-Tai Chang, Ping-Huai Wang, Mei-Yun Chang, Han-Shui Hsu

**Affiliations:** 1 Institute of Emergency and Critical Care Medicine, National Yang Ming Chiao Tung University, Taipei, Taiwan; 2 Department of Chest Medicine, Far Eastern Memorial Hospital, Taipei, Taiwan; 3 Department of Critical Care Medicine, Far Eastern Memorial Hospital, Taipei, Taiwan; 4 Department of Surgery, Taipei Veterans General Hospital, Taipei, Taiwan; Stanford University School of Medicine, UNITED STATES

## Abstract

The pathophysiology of coronavirus disease-2019 (COVID-19)-related acute respiratory distress syndrome (ARDS) varies from other pneumonia-related ARDS. We evaluated whether the mortality rates differed for COVID-19 and non-COVID-19-related ARDS in the Asian population in 2021. This single center retrospective observational cohort study included patients with COVID-19 and non-COVID-19-related ARDS that required invasive mechanical ventilation. The primary outcome was all-cause in-hospital mortality. The secondary outcomes included hospital length of stay, ICU length of stay, duration of mechanical ventilation, and ventilator-free days (VFDs) during the first 28 days. A 1:1 propensity score matching was performed to correct potential confounders by age, obesity or not, and ARDS severity. One-hundred-and-sixty-four patients fulfilled the inclusion criteria. After 1:1 propensity score matching, there were 50 patients in each group. The all-cause in-hospital mortality of all patients was 38 (38%), and no significant differences were found between COVID-19 and non-COVID-19-related ARDS (17 [34%) vs. 21 [42%], *p* = 0.410). Both groups had length of stay (30.0 [20.0–46.0] vs. 27.0 [13.0–45.0] days, *p* = 0.312), ICU length of stay (19.0 [13.0–35.0] vs. 16.0 [10.0–32.0] days, *p* = 0.249), length of mechanical ventilation (19.0 [10.0–36.0] vs. 14.0 [9.0–29.0] days, *p* = 0.488), and ventilator-free days during the first 28 days (5.5 [0.0–17.0] vs. 0.0 [0.0–14.0] days, *p* = 0.320). Immunocompromised status (Hazard ratio: 3.63; 95% CI: 1.51–8.74, *p* = 0.004) and progress to severe ARDS (Hazard ratio: 2.92; 95% CI: 1.18–7.22, *p* = 0.020) were significant in-hospital mortality-related confounders. There were no significant difference in mortality among both groups. Immunocompromised status and progression to severe ARDS are two possible risk factors for patients with ARDS; COVID-19 is not a mortality-related risk exposure.

## Introduction

Acute respiratory distress syndrome (ARDS) is a life-threatening severe lung inflammation [1], and pathophysiologic processes including increased pulmonary vascular permeability, leads to alveolar infiltration and ventilation/perfusion mismatch that can induce hypoxemic respiratory failure [2]. The LUNG SAFE study in 2016 investigated patients who developed ARDS in the first 48 hours and received invasive mechanical ventilation in Intensive Care Units (ICUs), and reported 40% hospital mortality in patients with ARDS, and 34.9%, 40.3%, 46.1% incidence for mild, moderate, and severe grades, respectively [2]. The severe acute respiratory syndrome coronavirus 2 (SARS-CoV-2) is a highly infectious RNA virus that causes coronavirus disease-2019 (COVID-19) and induces a critical viral pneumonia leading to ARDS. However, the respiratory physiology of COVID-19-related ARDS differs from other pneumonia-related ARDS with respect to lung compliance [3, 4] and ventilatory ratio (VR) [4–6], and its comorbidities such as diabetes mellitus, chronic renal failure, cardiovascular disease, asthma, chronic obstructive pulmonary disease (COPD), obesity, and immunocompromised status are potential risk factors [7]. We conducted a literature review to identify and compare the outcomes of COVID-19 and Non-COVID-19-related ARDS, globally [4, 8–16]; the mortality rates and source of databases varied several studies and most of studies conducted in 2020 focused on patients in the western countries. We hypothesized that in 2020, therapeutic management of COVID-19-related ARDS, including the effect of adjunctive respiratory therapy (prone positioning [17], recruitment maneuvers [8], inhaled nitric oxide [18], and extracorporeal membrane oxygenation [19]) and detailed planned medical treatments such as remdesivir [20] and dexamethasone [21], was not fully understood. Thus, this may have contributed to selection bias and confounders and resulted in different outcomes; furthermore, it is unclear whether there are other recent studies reporting different outcomes. Therefore, we aimed to evaluate whether the mortality rates differed among single center patients with COVID-19 and non-COVID-19-related ARDS that required invasive mechanical ventilation in Asian countries in 2021.

## Materials and methods

### Study population

This single center retrospective observational cohort study included patients with COVID-19 and non-COVID-19-related ARDS that required invasive mechanical ventilation. All patients with COVID-19 had positive real-time polymerase chain reaction (RT-PCR) results for severe acute respiratory syndrome coronavirus 2 (SARS-CoV-2) infection, and the diagnosis of ARDS was based on the Berlin definition. All patients with COVID-19 were unvaccinated. This study was approved by Research Ethics Review Committee of Far Eastern Memorial Hospital (FEMH No.111214-E), and requirement for informed consent form was waived due to the retrospective nature of the study.

### Data collection

Data between April 29, 2021 and August 3, 2021 for patients with COVID-19-related ARDS and January 1, 2021, and December 31, 2021 for patients with non-COVID-19-related ARDS were collected by reviewing the medical records in the Department of Critical Care Medicine, Far Eastern Memorial Hospital, Taipei, Taiwan.

The inclusion criteria were as follows: (1) age ≥18 years; (2) admitted to the medical ICU (MICU) between January 1, 2021 and December 31, 2021; (3) diagnosed with pneumonia; (4) required invasive mechanical ventilation; and (5) developed ARDS in the first 48 hours.

The exclusion criteria were as follows: (1) transferred to another hospital; (2) missing data; and (3) trauma.

The clinical characteristics obtained were as follows: age, sex, body mass index (BMI), and obesity defined as BMI ≥ 30 kg/m^2^; acute physiology and chronic health evaluation II scores and comorbidities including diabetes mellitus, chronic renal failure, cardiovascular disease, asthma, chronic obstructive pulmonary disease (COPD), and immunocompromised status; laboratory data such as leukocyte, neutrophil, lymphocyte, C-reactive protein (CRP), platelet, D-Dimer, and creatinine levels at the time of patient admission to the ICU.

“Symptoms for ICU admission day” was defined as the day from when patients with dyspnea, increased work of breathing or oxygen desaturation were admitted to the ICU. Adjunctive therapy including prone positioning, recruitment maneuvers, inhaled nitric oxide, and extracorporeal membrane oxygenation use during mechanical ventilation were recorded.

The severity of the ARDS was defined by the ratio of arterial oxygen tension to the fraction of inspired oxygen (PaO_2_/FiO_2_); this was classified as mild (200 <PaO_2_/FiO_2_ ≤300 mmHg), moderate (100 <PaO_2_/FiO_2_ ≤200 mmHg), and severe (PaO_2_/FiO_2_ ≤100 mmHg) based on the Berlin definition. PaO_2_/FiO_2_ ratio was collected within the first 48 hours after receiving invasive mechanical ventilation and followed-up.

The respiratory physiological parameters were collected when patients fulfilled the ARDS criteria with assist-control mode ventilation. The initial tidal volume (V_T_) was 6–10 mL/kg of predicted body weight (PBW) and positive end-expiratory pressure (PEEP) was set to a high FiO_2_/low PEEP table to maintain a plateau pressure (Pplat) ≤ 30 cmH2O and the ventilator was adjusted according to the lung protective ventilation strategy after patients fulfilled the ARDS criteria. Pplat was measured by inspiration-hold maneuver on the mechanical ventilator for 0.5–1 s. Driving pressure was calculated as the difference between Pplat and PEEP. Static compliance was the ratio of tidal volume to driving pressure. Mean airway pressure (Pmean) was calculated by the peak inspiratory pressure (PIP), PEEP, and inspiratory to expiratory time ratio. Ventilatory ratio (VR) was calculated as

VR=[Minuteventilation(ml/min)×PaCO2(mmHg)]÷(PBW×100×37.5).

The primary outcome was all-cause in-hospital mortality. The secondary outcomes included hospital length of stay, ICU length of stay, duration of mechanical ventilation, ventilator-free days (VFDs) during the first 28 days. VFD was considered as 0 if patients had died within 28 days.

### Statistical analysis

Continuous variables were presented as median (interquartile range) and compared with the Mann-Whitney U test. Categorical variables were presented as number (*n*) and proportion (%) and compared with the Chi-square or Fisher’s exact test. Kaplan-Meier survival curve was presented with cumulative incidence of 60-day mortality, and differences between COVID-19 and non-COVID-19-related ARDS were analyzed using the log-rank test.

A 1:1 propensity score matching was performed to correct potential confounders in the baseline characteristics by age, obesity or not, and ARDS severity; the univariate analysis results with *p*<0.1 was entered into the multivariate Cox proportional hazards model to identify risk factors influencing all-cause in-hospital mortality. A *p*-value < .05 was considered statistically significant. The statistical analyses were performed using SPSS 24.0 (IBM SPSS Statistics for Windows, Version 24.0, Armonk, NY: IBM Corp.). The protocol described in this article has been published in protocols.io (S1 Appendix).

## Results

### Study population and baseline characteristics

**Fig 1:**
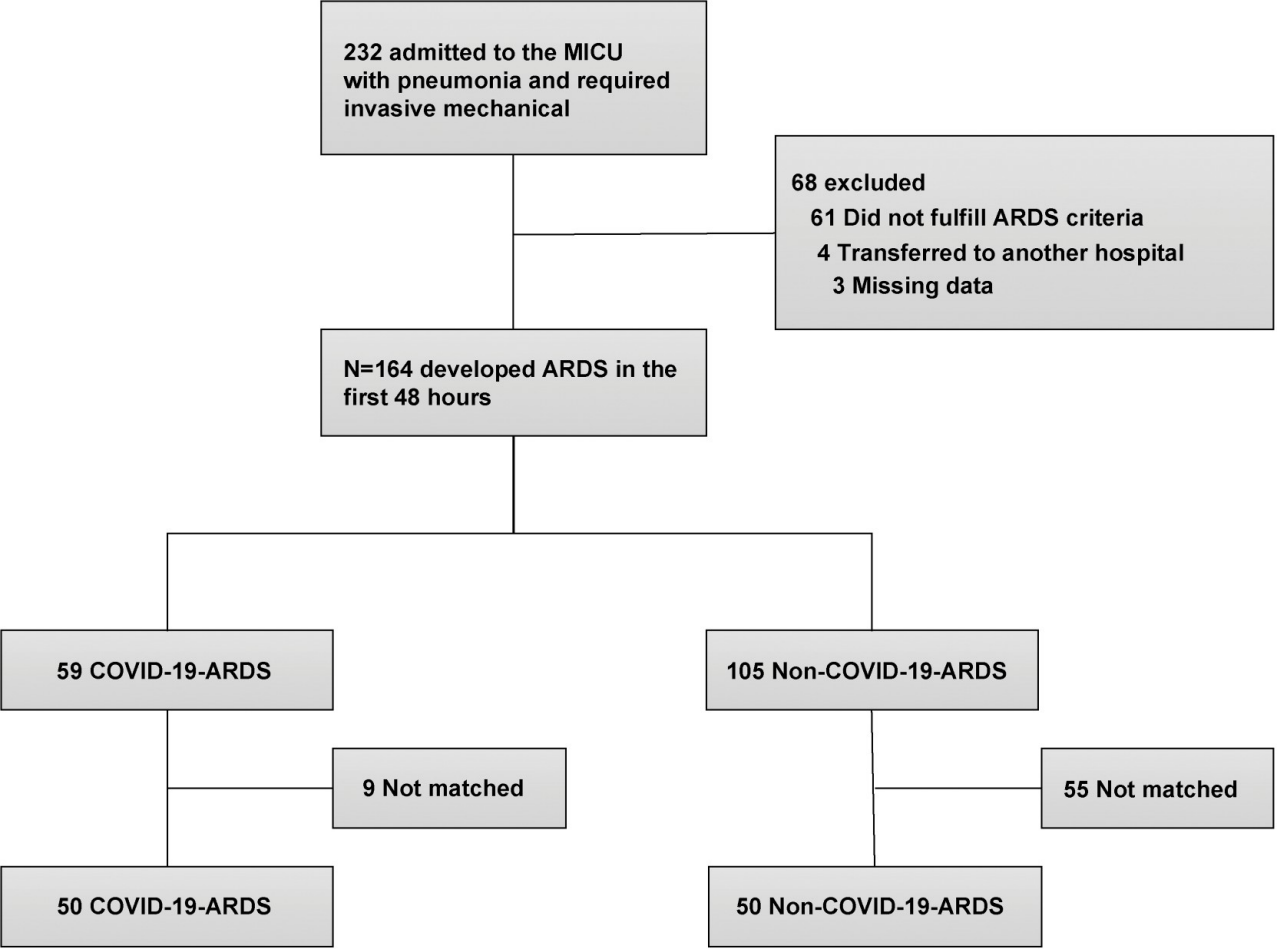
Flow chart of the screening process.

From January 1, 2021 to December 31, 2021, a total of 232 patients were admitted to the MICU of our hospital with pneumonia requiring invasive mechanical ventilation and 68 were excluded (61 did not fulfill the ARDS criteria, 4 were transferred to another hospital, and 3 had missing data). Finally, 164 patients were included and divided into 2 groups: COVID-19- related ARDS (59 patients) and non-COVID-19-related ARDS (105 patients). After 1:1 propensity score matching, there were 50 patients in each group (Fig 1).

Our results revealed that exposure to COVID-19 (Hazard ratio: 0.92; 95% confidence interval [CI]: 0.52–1.61, *p* = 0.760) and propensity score (Hazard ratio: 3.00; 95% CI: 0.37–24.33, *p* = 0.303) had no significant effect on mortality before propensity score matching. (S1 Table). The distribution of propensity score before and after matching is depicted in S1 Fig and all data before matching are presented in S2 and S3 Tables.

The baseline characteristics are presented in Table 1. The age (*p* = 0.497), proportion of the elderly aged >65 years (*p* = 0.673), obesity with BMI ≥ 30 kg/m^2^ (*p* = 0.505), and ARDS severity (*p* = 0.888) (Table 2) were balanced after propensity score matching; however, patients with COVID-19-related ARDS had higher BMI (25.6 [23.0–27.8] vs. 22.1 [19.5–24.2] kg/m^2^, *p*<0.001), lower APACHE II scores (20.5 [16.0–25.0] vs. 29.0 [25.0–33.0], *p*<0.001), and longer “symptoms for ICU admission days” (5.0 [2.0–7.0] vs. 1.0 [0.0–6.0] days, *p* = 0.001). The comorbidities of COVID-19-related ARDS were less chronic renal failure (2 [4.0%] vs. 10 [20.0%], *p* = 0.014), and immunocompromised status (0 [0.0%] vs. 10 [20.0%], *p* = 0.001), and laboratory data presenting higher neutrophil (88.5 [84.2–92.8] vs. 80.0 [54.0–89.4] %, *p*<0.001) and lower C-reactive protein (CRP) (9.4 [3.8–13.2] vs. 13.8 [8.4–21.9] mg/dL, *p* = 0.004), and creatinine (0.9 [0.7–1.1] vs. 1.3 [0.9–2.4], *p* = 0.001) levels.

**Table 1:** Baseline characteristics.

	All (*n* = 100)	COVID-19 ARDS (*n* = 50)	Non-COVID-19 ARDS (*n* = 50)	*p*-value
**Demographics**				
**Age, year**	68.0 (63.0–72.5)	68.0 (63.0–72.0)	69.5 (62.0–73.0)	0.497
**Age ≥ 65, n (%)**	66 (66.0)	34 (68.0)	32 (64.0)	0.673
**Male, n (%)**	70 (70.0)	34 (68.0)	36 (72.0)	0.663
**BMI^a^, kg/m^2^**	23.8 (21.2–26.5)	25.6 (23.0–27.8)	22.1 (19.5–24.2)	<0.001
**Obesity, n (%)**	10 (10.0)	4 (8.0)	6 (12.0)	0.505
**APACHE II^b^**	25.0 (21.2–26.5)	20.5 (16.0–25.0)	29.0 (25.0–33.0)	<0.001
**Symptom to ICU^c^, days**	3.5 (1.0–7.0)	5.0 (2.0–7.0)	1.0 (0.0–6.0)	0.001
**Comorbidity, n (%)**				
**Diabetes mellitus**	30 (30.0)	17 (34.0)	13 (26.0)	0.383
**Chronic renal failure**	12 (12.0)	2 (4.0)	10 (20.0)	0.014
**Cardiovascular disease**	29 (29.0)	12 (24.0)	17 (34.0)	0.271
**Asthma**	2 (2.0)	1 (2.0)	1 (2.0)	1.000
**COPD^d^**	9 (9.0)	2 (4.0)	7 (14.0)	0.160
**Immunocompromised**	10 (10.0)	0 (0.0)	10 (20.0)	0.001
**Laboratory data**				
**Leukocyte, 10^3^/μL**	10.5 (6.5–13.5)	9.8 (7.0–12.6)	11.0 (5.2–15.9)	0.647
**Neutrophil, %**	86.2 (75.8–91.5)	88.5 (84.2–92.8)	80.0 (54.0–89.4)	<0.001
**Lymphocyte, %**	6.4 (3.5–11.7)	6.1 (3.3–8.9)	7.3 (3.6–14.4)	0.102
**C-reactive protein, mg/dL**	11.7 (6.4–16.6)	9.4 (3.8–13.2)	13.8 (8.4–21.9)	0.004
**Platelet, 10^3^/μL**	182.0 (124.5–237.0)	182.5 (140.0–231.0)	182.0 (80.0–246.0)	0.759
**D-Dimer, μg/mL**	3.7 (1.5–10.0)	3.4 (1.1–10.0)	4.0 (1.7–9.7)	0.296
**Creatinine, mg/dL**	1.0 (0.8–1.7)	0.9 (0.7–1.1)	1.3 (0.9–2.4)	0.001

^a^ BMI, body mass index

^b^ APACHE II, acute physiology and chronic health evaluation

^c^ ICU, intensive care unit

^d^ COPD, chronic obstructive pulmonary disease.

**Table 2:** Respiratory physiology.

	All (*n* = 100)	COVID-19 ARDS (*n* = 50)	Non-COVID-19 ARDS (*n* = 50)	*p*-value
**ARDS^e^ severity, n (%)**				0.888
**Mild**	24 (24.0)	11 (22.0)	13 (26.0)	
**Moderate**	56 (56.0)	29 (58.0)	27 (54.0)	
**Severe**	20 (20.0)	10 (20.0)	10 (20.0)	
**Progression**				0.884
**Progress to moderate, n (%)**	13 (13.0)	6 (12.0)	7 (14.0)	
**Progress to severe, n (%)**	26 (26.0)	14 (28.0)	12 (24.0)	
**PaO2/FiO2 ratio, mmHg**	146.6 (106.4–197.7)	142.4 (105.2–189.4)	153.3 (116.2–210.8)	0.198
**PaO2, mmHg**	127.7 (89.4–181.6)	132.9 (88.4–186.0)	126.6 (89.9–179.7)	0.931
**FiO2, %**	100.0 (80.0–100.0)	100.0 (85.0–100.0)	100.0 (80.0–100.0)	0.287
**pH**	7.36 (7.32–7.44)	7.37 (7.32–7.44)	7.36 (7.33–7.44)	0.684
**PaCO2, mmHg**	39.2 (33.7–44.6)	39.4 (34.5–43.5)	37.9 (33.5–44.7)	0.899
**V_T_, ml/kg PBW^f^**	7.87 (7.08–8.70)	7.63 (7.05–8.48)	7.96 (7.08–8.79)	0.277
**Minute ventilation, L/min**	9.1 (6.9–10.9)	8.5 (6.7–10.3)	9.3 (7.9–11.6)	0.110
**PEEP^g^, cmH2O**	10.0 (8.0–12.0)	10.0 (8.0–12.0)	8.0 (8.0–10.0)	0.008
**Plateau pressure, cmH2O**	24.0 (21.0–28.0)	26.0 (22.0–30.0)	22.0 (20.0–26.0)	0.005
**Driving pressure, cmH2O**	15.0 (13.0–18.4)	15.0 (13.0–20.0)	14.5 (12.0–18.0)	0.511
**Static compliance, ml/cmH2O**	34.0 (25.5–43.0)	32.2 (24.0–38.5)	35.2 (27.0–44.8)	0.082
**Mean airway pressure, cmH2O**	14.0 (12.0–16.1)	15.0 (13.0–16.9)	13.5 (11.0–16.0)	0.060
**Ventilatory ratio**	1.61 (1.32–1.95)	1.53 (1.23–1.85)	1.74 (1.38–2.07)	0.052
**Adjunctive therapy, n (%)**				
**Prone positioning**	34 (34.0)	29 (58.0)	5 (10.0)	<0.001
**Recruitment maneuvers**	22 (22.0)	18 (36.0)	4 (8.0)	0.001
**Inhaled Nitric Oxide**	8 (8.0)	0 (0.0)	8 (16.0)	0.006
**ECMO^h^**	2 (2.0)	1 (2.0)	1 (2.0)	1.000

^e^ ARDS, acute respiratory distress syndrome

^f^ PBW, predicted body weight

^g^ PEEP, positive end-expiratory pressure

^h^ ECMO, extracorporeal membrane oxygenation

### Respiratory physiology

All respiratory physiologic parameters were adequately balanced, except higher initial PEEP (10.0 [8.0–12.0] vs. 8.0 [8.0–10.0] cmH2O, *p* = 0.008) and adjunctive therapies that presented with higher prone positioning (29 [58.0%] vs. 5 [10.0%], *p*<0.001) and recruitment maneuvers (18 [36.0%] vs. 4 [8.0%], *p* = 0.001), and lower inhaled nitric oxide (0 [0.0%] vs. 8 [16.0%], *p* = 0.006) proportions (Table 2).

### Outcomes

The all-cause in-hospital mortality of all patients was 38 (38%), and no significant differences were found between COVID-19 and non-COVID-19-related ARDS (17 [34%) vs. 21 [42%], *p* = 0.410). Hospital length of stay (30.0 [20.0–46.0] vs. 27.0 [13.0–45.0] days, *p* = 0.312), ICU length of stay (19.0 [13.0–35.0] vs. 16.0 [10.0–32.0] days, *p* = 0.249), duration of mechanical ventilation (19.0 [10.0–36.0] vs. 14.0 [9.0–29.0] days, *p* = 0.488), and ventilator-free days during the first 28 days (5.5 [0.0–17.0] vs. 0.0 [0.0–14.0] days, *p* = 0.320) were similar in both groups (Table 3).

**Table 3:** Outcomes.

	All (*n* = 100)	COVID-19 ARDS (*n* = 50)	Non-COVID-19 ARDS (*n* = 50)	*p*-value
**Primary outcome**				
**Hospital mortality, n (%)**	38 (38.0)	17 (34.0)	21 (42.0)	0.410
**Secondary outcome**				
**Hospital length of stay, days**	28.0 (17.0–46.0)	30.0 (20.0–46.0)	27.0 (13.0–45.0)	0.312
**ICU length of stay, days**	17.0 (12.0–33.5)	19.0 (13.0–35.0)	16.0 (10.0–32.0)	0.249
**Length of MV^i^, days**	15.0 (9.0–31.5)	19.0 (10.0–36.0)	14.0 (9.0–29.0)	0.488
**VFD^j^ at 28 days, days**	0.5 (0.0–17.0)	5.5 (0.0–17.0)	0.0 (0.0–14.0)	0.320

^i^ MV, mechanical ventilation

^j^ VFD, ventilator-free days

We analyzed the risk factors associated with in-hospital mortality for all patients using the Cox proportional hazards model and entered the variables of immunocompromised status (*p* = 0.001), severe ARDS (*p* = 0.041), progress to moderate ARDS (*p* = 0.041), progress to severe ARDS (*p* = 0.013), and static compliance (*p* = 0.094) from univariate analysis to multivariate analysis, which revealed that immunocompromised status (Hazard ratio: 3.63; 95% CI: 1.51–8.74, *p* = 0.004) and progress to severe ARDS (Hazard ratio: 2.92; 95% CI: 1.18–7.22, *p* = 0.020) were significant confounders related to in-hospital mortality.

In addition, COVID-19 (*p* = 0.346), age (*p* = 0.380), BMI (*p* = 0.568), APACHE II scores (*p* = 0.160), and chronic renal failure (*p* = 0.632), and plateau pressure (*p* = 0.324) was not significant risk factors in any of the patients with ARDS (Table 4).

**Table 4:** Risk factors associated with hospital mortality of all patients (Cox proportional hazards model).

	Univariate analysis		Multivariable analysis	
**Predictive variables**	**HR (95% CI)**	***P* value**	**HR (95% CI)**	***P* value**
**Age, yr**	0.99 (0.96–1.02)	0.380		
**Male, n (%)**	0.73 (0.37–1.46)	0.370		
**BMI, kg/m^2^**	0.98 (0.93–1.04)	0.568		
**APACHE II**	1.03 (0.99–1.07)	0.160		
**COVID-19^k^**	0.74 (0.39–1.40)	0.346		
**Comorbidity, n (%)**				
**Diabetes mellitus**	0.70 (0.34–1.45)	0.341		
**Chronic renal failure**	0.78 (0.28–2.19)	0.632		
**Cardiovascular disease**	0.90 (0.44–1.81)	0.759		
**Asthma**	0.05 (0.00–261.83)	0.632		
**COPD**	0.67 (0.20–2.19)	0.504		
**Immunocompromised**	3.96 (1.79–8.77)	0.001	3.63 (1.51–8.74)	0.004
**Respiratory physiology**				
**ARDS severity, n (%)**				
**Mild**	Reference			
**Moderate**	1.53 (0.65–3.61)	0.329		
**Severe**	2.76 (1.04–7.32)	0.041	2.04 (0.59–7.06)	0.260
**Progression**				
**Progress to moderate, n (%)**	0.17 (0.02–1.28)	0.085	0.18 (0.02–1.71)	0.135
**Progress to severe, n (%)**	2.28 (1.19–4.39)	0.013	2.92 (1.18–7.22)	0.020
**V_T_, ml/kg PBW**	1.00 (0.80–1.25)	0.984		
**Plateau pressure, cmH2O**	1.03 (0.97–1.08)	0.324		
**Static compliance, ml/cmH2O**	0.98 (0.95–1.00)	0.094	0.99 (0.95–1.02)	0.374
**Ventilatory ratio**	1.27 (0.75–2.14)	0.369		

^k^ COVID-19, coronavirus disease 19

**Fig 2:**
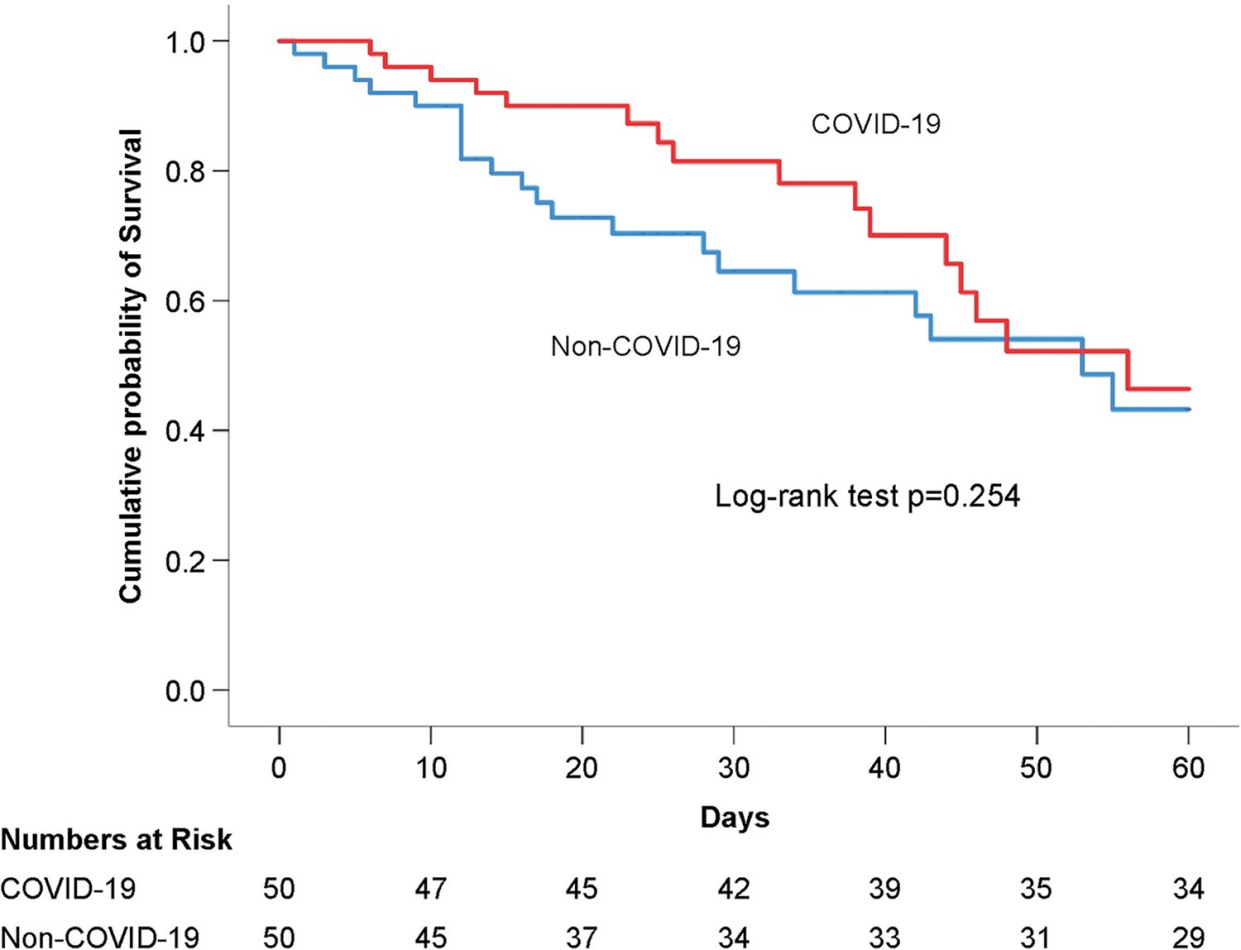
Kaplan-Meier survival curve of COVID-19 and non-COVID-19-related ARDS.

In Kaplan-Meier survival curve based on the 60-day in-hospital mortality rates, there was no significant difference between both groups (mean survival time: 46.6; 95% CI: 41.3–51.9 vs. 41.1; 95% CI: 34.8–47.4 days; log-rank test *p* = 0.254) (Fig 2).

Subgroup analysis showed that the in-hospital mortality rate did not differ significantly between COVID-19 ARDS patients with or without bacterial infections (9 [34.6%] vs. 8 [33.3%], p = 0.924). Moreover, length of stay (37.0 [21.0–58.0] vs. 25.5 [19.5–40.5] days, p = 0.084), and VFD at 28 days (0.0 [0.0–17.0] vs. 8.5 [0.0–19.5] days, p = 0.161) were similar in both groups. While, the ICU length of stay (25.0 [15.0–39.0] vs. 15.0 [10.0–26.5] days, p = 0.021) and length of MV (22.0 [11.0–44.0] vs. 13.0 [8.0–23.5] days, p = 0.039) were longer in patients with bacterial infections (Table 5).

**Table 5:** Outcomes of COVID-19 ARDS with or without bacterial infections.

	All (*n* = 50)	COVID-19 ARDS with bacteria infection (*n* = 26)	COVID-19 ARDS without bacteria infection (*n* = 24)	*p*-value
**Hospital mortality, n (%)**	17 (34.0)	9 (34.6)	8 (33.3)	0.924
**Hospital length of stay, days**	30.0 (20.0–46.0)	37.0 (21.0–58.0)	25.5 (19.5–40.5)	0.084
**ICU length of stay, days**	19.0 (13.0–35.0)	25.0 (15.0–39.0)	15.0 (10.0–26.5)	0.021
**Length of MV^i^, days**	19.0 (10.0–36.0)	22.0 (11.0–44.0)	13.0 (8.0–23.5)	0.039
**VFD^j^ at 28 days, days**	5.5 (0.0–17.0)	0.0 (0.0–17.0)	8.5 (0.0–19.5)	0.161

^i^ MV, mechanical ventilation

^j^ VFD, ventilator-free days

## Discussion

Globally, after the COVID-19 pandemic, treatment protocols have been constantly updated based on new research. Previous studies reported different mortality rates for COVID-19 and non-COVID-19-related ARDS across countries, and most studies conducted in 2020 included patients from the western countries, except some studies that included control groups from pre-COVID-19 databases [4, 8–15]. To the best of our knowledge, this is the first single-center retrospective observational cohort study that compares the mortality of COVID-19 and non-COVID-19-related ARDS that required invasive mechanical ventilator in Asian countries in 2021. In addition, the SARS-CoV-2 in this study period is characterized by a predominance of the Delta variant. Treatments were based on updated international protocols.

Older age, obesity [7, 22, 23], and ARDS severity were the three risk factors associated with mortality in baseline characteristics, and the different distribution could produce potential confounders; therefore, propensity score matching to eliminate the confounding factors and additionally, regression analyses were performed. Hence, ARDS severity and its progression and PaO_2_/FiO_2_ to classify the grade of disease were elucidated. Both groups were equally distributed. Despite a significantly higher BMI, lower APACHE II scores, and less chronic renal failure in patients with COVID-19-related ARDS, these confounders did not influence mortality rates in univariate analysis.

There are several findings in our study. First, the all-cause in-hospital mortality rates in both groups are not significantly different, and the results are consistent with those reported in several published studies [14]. Furthermore, the results of COVID-19 and non-COVID-19-related ARDS mortality rates are similar with LUNG SAFE study [2]; however, the hospital length of stay, ICU length of stay, duration of mechanical ventilation, and ventilator-free days during the first 28 days are longer. Second, we chose the 60-day Kaplan-Meier survival curve close to the hospital length of stay in our study population for time to event analysis, and there was no significant difference between COVID-19 and non-COVID-19-related ARDS groups. Third, owing to similar outcomes in both groups, all ARDS patients were included and COVID-19 was independently added to the univariate analysis. Fourth, severity and not risk exposure, and progress of ARDS and immunocompromised status were observed to cause pneumonia due to a poor immune system and induce ARDS resulting in death, especially in patients with non-COVID-19-related ARDS. Consequently, the subgroup of COVID-19 combined with bacterial associated ARDS probably required additional more time of due to infection control which could account for the increased ICU length of stay and duration of mechanical ventilation.

In our study population, a shorter “symptoms for ICU admission day” for non-COVID-19-related ARDS was observed, which indicates a more rapid disease progression than that reported in other studies [16]. All patients developing a new severe respiratory symptom within 1 week, according to the ARDS criteria [1], were admitted to the ICU and moderate severity was considered. We set a higher initial PEEP in COVID-19-related ARDS to reduce the frequency of adjusting and patient contact time to protect medical staff; however, we adjusted it according to the clinical condition afterward and maintained lung protective strategy to prevent Pplat >30 cmH2O [24]. In addition, the compliance of both groups were not different in statistical analysis and are similar to that reported in previous studies; however, some of these studies described a relatively normal respiratory system compliance in COVID-19-related ARDS that was higher at day 1 though not at day 3, when compared with non-COVID-19-related ARDS. Hence, we could not compare compliance change over time because the data were collected within the first 48 hours and were lost to follow-up [4].

VR, calculated by the aforementioned formula, is used for dead space evaluation and it is affected by humidification devices such as heat and moisture exchangers or heated humidifiers. An approximate VR value of 1 represents normal lung ventilation and increase in VR may be accompanied by deteriorating gas exchange that leads to higher PaCO_2_ [5, 6]. The patients with COVID-19 and non-COVID-19 included in our study were ventilated with only heated humidifiers and demonstrated nearly consistent results of VR and PaCO_2_. Adjunctive therapies in ARDS have been necessary to improve oxygenation [25] and mortality [26–28], and research on using these therapies in COVID-19-related ARDS are constantly updated. We assumed that COVID-19 was similar to classical ARDS in our study population; thus, prone positioning and recruitment maneuvers should be performed under stable hemodynamic conditions. We observed that lower percentage of prone positioning and recruitment maneuvers, and higher percentage of inhaled nitric oxide in non-COVID-19-related ARDS were due to comorbidities causing complications or contraindications such as septic shock or other unstable hemodynamic conditions; however, this has not been reported in the present study. In cases of a potential negative impact on hemodynamics, inhaled nitric oxide may be used to dilate non-perfused pulmonary vessel to reduce the dead space and further improve PaO2/FiO2.

Our study has several limitations. First, since this is a single-center retrospective observational cohort study, there are other possible prognosis-related variables related that may cause selection bias, except in cases of COVID-19; however, we have included participants from both groups at the same time, of the same race, and from the same hospital to eliminate some of the potential confounders. Second, the study population included a small sample size. Therefore, the clinical outcomes represent only the patients with ARDS in Asian countries. Although we have performed a propensity score matching to eliminate patients with extreme values, we have only adjusted for known potential confounders and may have missed the others. Moreover, a few unbalanced variables remained; thus, we further confirmed the effect of disturbance using the Cox proportional hazards model. Third, in COVID-19, pulmonary embolism is a complication with higher D-Dimer values [29]; however, we did not obtain its in-hospital diagnostic data after the patients were included because the data was collected at the time of ARDS diagnosis; however, we compared the initial D-Dimer values and it revealed balanced result regardless of COVID-19 exposure. Fourth, in our study, all patients with COVID-19-related ARDS were unvaccinated; this was possibly because of the vaccination policy, incomplete information, and people’s choices in 2021; thus, the result of study represents only unvaccinated patients. Fifth, in our study, the exposure of patients with ARDS and without COVID-19 were inconsistent because most of the results of sputum culture were either bacterial pneumonia or no growth, except one with cytomegalovirus-related pneumonia.

## Conclusion

In this single center, retrospective, observational cohort study including unvaccinated patients with COVID-19 and non-COVID-19-related ARDS that required invasive mechanical ventilation, no significant differences were observed in mortality rates between both groups, and these results are consistent with those reported in previous studies. Immunocompromised status and progress to severe ARDS are two possible risk factors among patients with ARDS, and COVID-19 is not a mortality-related risk exposure. Globally, vaccination coverage and treatment guidelines will continue to be updated in line with new research. A multicenter prospective observational cohort study with a large sample size and rigorous study design would increase the power of the research and eliminate the confounders reported in this study.

## Supporting information

S1 Appendix Study protocols. (DOCX)

S1 Fig Histogram of propensity score before and after matching. (DOCX)

S1 Table Propensity score Hazard ratio. (DOCX)

S2 Table Baseline characteristics before propensity score matching. (DOCX)

S3 Table Respiratory physiology before propensity score matching. (DOCX)

## References

[1] Force ADT, Ranieri VM, Rubenfeld GD, Thompson BT, Ferguson ND, Caldwell E, et al. Acute respiratory distress syndrome: the Berlin Definition. JAMA. 2012;307(23):2526–2533. doi: 10.1001/jama.2012.5669 22797452

[2] Bellani G, Laffey JG, Pham T, Fan E, Brochard L, Esteban A, et al. Epidemiology, patterns of care, and mortality for patients with acute respiratory distress syndrome in intensive care units in 50 countries. JAMA. 2016;315(8):788–800. doi: 10.1001/jama.2016.0291 26903337

[3] Mercat A, Richard JCM, Vielle B, Jaber S, Osman D, Diehl JL, et al. Positive end-expiratory pressure setting in adults with acute lung injury and acute respiratory distress syndrome: a randomized controlled trial. JAMA. 2008;299(6):646–655. doi: 10.1001/jama.299.6.646 18270353

[4] Beloncle F, Studer A, Seegers V, Richard JC, Desprez C, Fage N, et al. Longitudinal changes in compliance, oxygenation and ventilatory ratio in COVID-19 versus non-COVID-19 pulmonary acute respiratory distress syndrome. Crit Care. 2021;25(1):248. doi: 10.1186/s13054-021-03665-8 34266454 PMC8280689

[5] Sinha P, Calfee CS, Beitler JR, Soni N, Ho K, Matthay MA, et al. Physiologic analysis and clinical performance of the ventilatory ratio in acute respiratory distress syndrome. Am J Respir Crit Care Med. 2019;199(3):333–341. doi: 10.1164/rccm.201804-0692OC 30211618 PMC6363976

[6] Liu X, Liu X, Xu Y, Xu Z, Huang Y, Chen S, et al. Ventilatory ratio in hypercapnic mechanically ventilated patients with Covid-19-associated acute respiratory distress syndrome. Am J Respir Crit Care Med. 2020;201(10):1297–1299. doi: 10.1164/rccm.202002-0373LE 32203672 PMC7233337

[7] Garg S, Kim L, Whitaker M, O’Halloran A, Cummings C, Holstein R, et al. Hospitalization rates and characteristics of patients hospitalized with laboratory-confirmed coronavirus disease 2019—COVID-NET, 14 States, March 1–30, 2020. MMWR Morb Mortal Wkly Rep. 2020;69(15):458–464. doi: 10.15585/mmwr.mm6915e3 32298251 PMC7755063

[8] Brault C, Zerbib Y, Kontar L, Fouquet U, Carpentier M, Metzelard M, et al. COVID-19- versus non-COVID-19-related acute respiratory distress syndrome: differences and similarities. Am J Respir Crit Care Med. 2020;202(9):1301–1304. doi: 10.1164/rccm.202005-2025LE 32857595 PMC7605202

[9] Chiumello D, Busana M, Coppola S, Romitti F, Formenti P, Bonifazi M, et al. Physiological and quantitative CT-scan characterization of COVID-19 and typical ARDS: a matched cohort study. Intensive Care Med. 2020;46(12):2187–2196. doi: 10.1007/s00134-020-06281-2 33089348 PMC7577365

[10] Ferrando C, Suarez-Sipmann F, Mellado-Artigas R, Hernandez M, Gea A, Arruti E, et al. Clinical features, ventilatory management, and outcome of ARDS caused by COVID-19 are similar to other causes of ARDS. Intensive Care Med. 2020;46(12):2200–2211. doi: 10.1007/s00134-020-06192-2 32728965 PMC7387884

[11] Grasselli G, Tonetti T, Protti A, Langer T, Girardis M, Bellani G, et al. Pathophysiology of COVID-19-associated acute respiratory distress syndrome: a multicentre prospective observational study. The Lancet Respiratory Medicine. 2020;8(12):1201–8. doi: 10.1016/S2213-2600(20)30370-2 32861276 PMC7834127

[12] Wahidi MM, Lamb C, Murgu S, Musani A, Shojaee S, Sachdeva A, et al. American Association for Bronchology and Interventional Pulmonology (AABIP) Statement on the use of bronchoscopy and respiratory specimen collection in patients with suspected or confirmed COVID-19 infection. J Bronchology Interv Pulmonol. 2020;27(4):e52–e4. doi: 10.1097/LBR.0000000000000681 32195687 PMC7141581

[13] Bain W, Yang H, Shah FA, Suber T, Drohan C, Al-Yousif N, et al. COVID-19 versus non-COVID-19 acute respiratory distress syndrome: comparison of demographics, physiologic parameters, inflammatory biomarkers, and clinical outcomes. Ann Am Thorac Soc. 2021;18(7):1202–1210. doi: 10.1513/AnnalsATS.202008-1026OC 33544045 PMC8328355

[14] Dmytriw AA, Chibbar R, Chen PPY, Traynor MD, Kim DW, Bruno FP, et al. Outcomes of acute respiratory distress syndrome in COVID-19 patients compared to the general population: a systematic review and meta-analysis. Expert Rev Respir Med. 2021;15(10):1347–1354. doi: 10.1080/17476348.2021.1920927 33882768 PMC8108193

[15] Sjoding MW, Admon AJ, Saha AK, Kay SG, Brown CA, Co I, et al. Comparing clinical features and outcomes in mechanically ventilated patients with COVID-19 and acute respiratory distress syndrome. Ann Am Thorac Soc. 2021;18(11):1876–1885. doi: 10.1513/AnnalsATS.202008-1076OC 33577740 PMC8641825

[16] Kutsogiannis DJ, Alharthy A, Balhamar A, Faqihi F, Papanikolaou J, Alqahtani SA, et al. Mortality and pulmonary embolism in acute respiratory distress syndrome from COVID-19 vs. non-COVID-19. Front Med (Lausanne). 2022;9:800241. doi: 10.3389/fmed.2022.800241 35308552 PMC8931188

[17] Weiss TT, Cerda F, Scott JB, Kaur R, Sungurlu S, Mirza SH, et al. Prone positioning for patients intubated for severe acute respiratory distress syndrome (ARDS) secondary to COVID-19: a retrospective observational cohort study. Br J Anaesth. 2021;126(1):48–55. doi: 10.1016/j.bja.2020.09.042 33158500 PMC7547633

[18] Garfield B, McFadyen C, Briar C, Bleakley C, Vlachou A, Baldwin M, et al. Potential for personalised application of inhaled nitric oxide in COVID-19 pneumonia. Br J Anaesth. 2021;126(2):e72–e75. doi: 10.1016/j.bja.2020.11.006 33288208 PMC7666572

[19] Garfield B, Bianchi P, Arachchillage D, Hartley P, Naruka V, Shroff D, et al. Six month mortality in patients with COVID-19 and non-COVID-19 viral pneumonitis managed with veno-venous extracorporeal membrane oxygenation. ASAIO J. 2021;67(9):982–988. doi: 10.1097/MAT.0000000000001527 34144551

[20] Beigel JH, Tomashek KM, Dodd LE, Mehta AK, Zingman BS, Kalil AC, et al. Remdesivir for the treatment of Covid-19—final report. N Engl J Med. 2020;383(19):1813–1826. doi: 10.1056/NEJMoa2007764 32445440 PMC7262788

[21] RECOVERY Collaborative Group, Horby P, Lim WS, Emberson JR, Mafham M, Bell JL, et al. Dexamethasone in hospitalized patients with Covid-19. N Engl J Med. 2021;384(8):693–704. doi: 10.1056/NEJMoa2021436 32678530 PMC7383595

[22] Kompaniyets L, Goodman AB, Belay B, Freedman DS, Sucosky MS, Lange SJ, et al. Body mass index and risk for COVID-19-related hospitalization, intensive care unit admission, invasive mechanical ventilation, and death—United States, March-December 2020. MMWR Morb Mortal Wkly Rep. 2021;70(10):355–361. doi: 10.15585/mmwr.mm7010e4 33705371 PMC7951819

[23] Tolossa T, Merdassa Atomssa E, Fetensa G, Bayisa L, Ayala D, Turi E, et al. Acute respiratory distress syndrome among patients with severe COVID-19 admitted to treatment center of Wollega University Referral Hospital, Western Ethiopia. PLoS One. 2022;17(6):e0267835. doi: 10.1371/journal.pone.0267835 35709142 PMC9202843

[24] Brower RG, Matthay MA, Morris A, Schoenfeld D, Thompson BT, Wheeler A. Ventilation with lower tidal volumes as compared with traditional tidal volumes for acute lung injury and the acute respiratory distress syndrome. N Engl J Med. 2000;342(18):1301–1308. doi: 10.1056/NEJM200005043421801 10793162

[25] Gebistorf F, Karam O, Wetterslev J, Afshari A. Inhaled nitric oxide for acute respiratory distress syndrome (ARDS) in children and adults. Cochrane Database Syst Rev. 2016;2016(6):CD002787. doi: 10.1002/14651858.CD002787.pub3 27347773 PMC6464789

[26] Guerin C, Reignier J, Richard JC, Beuret P, Gacouin A, Boulain T, et al. Prone positioning in severe acute respiratory distress syndrome. N Engl J Med. 2013;368(23):2159–2168. doi: 10.1056/NEJMoa1214103 23688302

[27] Hodgson C, Goligher EC, Young ME, Keating JL, Holland AE, Romero L, et al. Recruitment manoeuvres for adults with acute respiratory distress syndrome receiving mechanical ventilation. Cochrane Database Syst Rev. 2016;11(11):CD006667. doi: 10.1002/14651858.CD006667.pub3 27855477 PMC6464835

[28] Munshi L, Walkey A, Goligher E, Pham T, Uleryk EM, Fan E. Venovenous extracorporeal membrane oxygenation for acute respiratory distress syndrome: a systematic review and meta-analysis. The Lancet Respiratory Medicine. 2019;7(2):163–172. doi: 10.1016/S2213-2600(18)30452-1 30642776

[29] Kwee RM, Adams HJA, Kwee TC. Pulmonary embolism in patients with COVID-19 and value of D-dimer assessment: a meta-analysis. Eur Radiol. 2021;31(11):8168–8186. doi: 10.1007/s00330-021-08003-8 33966132 PMC8106765

