# Supplementary material for: Mortality in patients with COVID-19 versus non-COVID-19- related acute respiratory distress syndrome: A single center retrospective observational cohort study
Source: PLoS One. 2023 Jun 2;18(6):e0286564. doi: 10.1371/journal.pone.0286564 (PMC10237657; doi:10.1371/journal.pone.0286564)
Supplement: S1 Appendix — (DOCX) [file pone.0286564.s001.docx]

**S1 Appendix. Study protocols**

Doi: [dx.doi.org/10.17504/protocols.io.bp2l6961rlqe/v2](https://dx.doi.org/10.17504/protocols.io.bp2l6961rlqe/v2)
