# Supplementary figures and images for: Mortality in patients with COVID-19 versus non-COVID-19- related acute respiratory distress syndrome: A single center retrospective observational cohort study
Source: PLoS One. 2023 Jun 2;18(6):e0286564. doi: 10.1371/journal.pone.0286564 (PMC10237657; doi:10.1371/journal.pone.0286564)

**
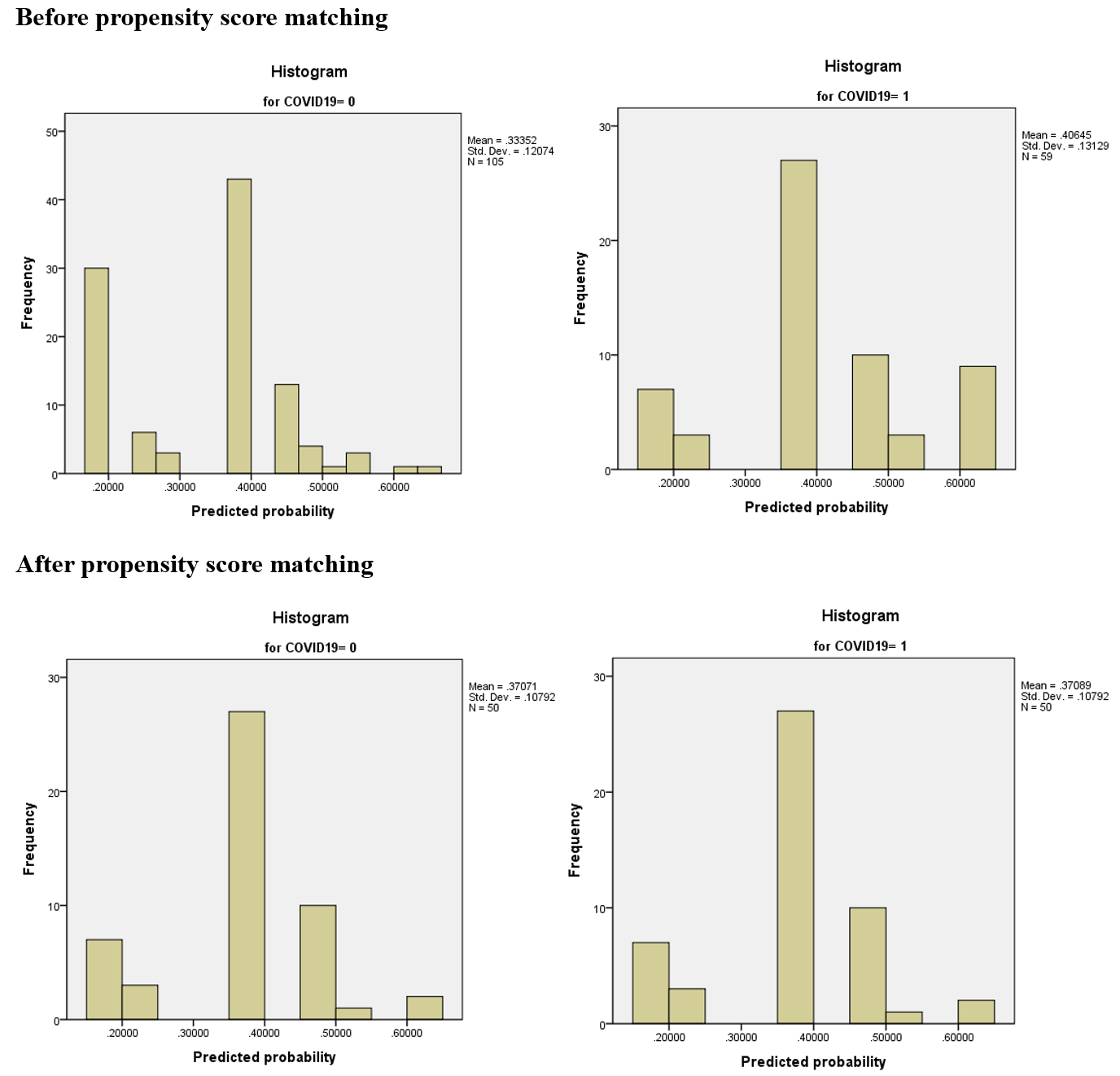
**

**S1 Figure**. Histogram of propensity score before and after matching

Supplement: S1 Fig — (DOCX) [file pone.0286564.s002.docx]
