# Supplementary material for: Mortality in patients with COVID-19 versus non-COVID-19- related acute respiratory distress syndrome: A single center retrospective observational cohort study
Source: PLoS One. 2023 Jun 2;18(6):e0286564. doi: 10.1371/journal.pone.0286564 (PMC10237657; doi:10.1371/journal.pone.0286564)
Supplement: S1 Table — (DOCX) [file pone.0286564.s003.docx]

**S1 Table**. Propensity score Hazard Ratio

| Predictive variables | HR (95% CI) | *p*-value |
| --- | --- | --- |
| COVID-19 | 0.92 (0.52–1.61) | 0.760 |
| Propensity score | 3.00 (0.37–24.33) | 0.303 |
