# Supplementary material for: Mortality in patients with COVID-19 versus non-COVID-19- related acute respiratory distress syndrome: A single center retrospective observational cohort study
Source: PLoS One. 2023 Jun 2;18(6):e0286564. doi: 10.1371/journal.pone.0286564 (PMC10237657; doi:10.1371/journal.pone.0286564)
Supplement: S2 Table — (DOCX) [file pone.0286564.s004.docx]

**S2 Table**. Baseline characteristics before propensity score matching

|  | All  (*n*=164) | COVID-19 ARDS  (*n*=59) | Non-COVID-19 ARDS  (*n*=105) | *p*-value |
| --- | --- | --- | --- | --- |
| Demographics | | | | |
| Age, year | 69.0 (63.5–74.5) | 67.0 (62.0–72.0) | 70.0 (64.0–78.0) | 0.014 |
| Age ≥ 65, n (%) | 113 (68.9) | 36 (61.0) | 77 (73.3) | 0.102 |
| Male, n (%) | 55 (33.5) | 41 (69.5) | 68 (64.8) | 0.538 |
| BMI, kg/m^2^ | 23.9 (20.8–27.2) | 26.1 (23.7–29.2) | 22.6 (19.5–24.6) | <0.001 |
| Obesity, n (%) | 25 (15.2) | 13 (22.0) | 12 (11.4) | 0.070 |
| APACHE II | 25.0 (20.0–30.5) | 20.0 (16.5–24.5) | 27.0 (24.0–32.0) | <0.001 |
| Symptom to ICU, days | 2.0 (1.0–7.0) | 5.0 (2.0–7.0) | 1.0 (0.0–6.0) | <0.001 |
| Comorbidity, n (%) | | | | |
| Diabetes mellitus | 59 (36.0) | 21 (35.6) | 38 (36.2) | 0.939 |
| Chronic renal failure | 34 (20.7) | 4 (6.8) | 30 (28.6) | 0.001 |
| Cardiovascular disease | 57 (34.8) | 13 (22.0) | 44 (41.9) | 0.010 |
| Asthma | 4 (2.4) | 1 (1.7) | 3 (2.9) | 1.000 |
| COPD | 18 (11.0) | 2 (3.4) | 16 (15.2) | 0.020 |
| Immunocompromised | 14 (8.5) | 0 (0.0) | 14 (13.3) | 0.003 |
| Laboratory data | | | | |
| Leukocyte, 10^3^/μL | 11.2 (9.1–13.6) | 9.6 (7.1–12.7) | 12.1 (7.7–16.7) | 0.033 |
| Neutrophil, % | 86.5 (79.1–94.6) | 89.4 (84.2–92.1) | 85.0 (72.1–91.0) | 0.003 |
| Lymphocyte, % | 6.4 (3.6–10.3) | 6.4(4.0–8.7) | 6.7 (3.3–11.0) | 0.617 |
| C-reactive protein, mg/dL | 11.7 (3.8–17.8) | 8.6 (2.4–13.1) | 13.8 (5.7–21.9) | <0.001 |
| Platelet, 10^3^/μL | 192.5 (127.0–266.5) | 190 (143.5–231.5) | 200.0 (110.0–270.0) | 0.99 |
| D-Dimer, μg/mL | 3.7 (1.6–9.5) | 2.6 (1.2–9.7) | 4.0 (1.8–8.4) | 0.134 |
| Creatinine, mg/dL | 1.1 (0.8–1.8) | 0.9 (0.7–1.2) | 1.3 (0.9–2.3) | <0.001 |

^a^ BMI, body mass index; ^b^ APACHE II, acute physiology and chronic health evaluation; ^c^ ICU, intensive care unit; ^d^ COPD, chronic obstructive pulmonary disease
