# Supplementary material for: Mortality in patients with COVID-19 versus non-COVID-19- related acute respiratory distress syndrome: A single center retrospective observational cohort study
Source: PLoS One. 2023 Jun 2;18(6):e0286564. doi: 10.1371/journal.pone.0286564 (PMC10237657; doi:10.1371/journal.pone.0286564)
Supplement: S3 Table — (DOCX) [file pone.0286564.s005.docx]

**S3 Table**. Respiratory physiology before propensity score matching

|  | All  (*n*=164) | COVID-19 ARDS  (*n*=59) | Non-COVID-19 ARDS  (*n*=105) | *p*-value |
| --- | --- | --- | --- | --- |
| ARDS severity, n (%) |  |  |  | 0.016 |
| Mild | 53 (32.3) | 11 (18.6) | 42 (40.0) |  |
| Moderate | 84 (51.2) | 35 (59.3) | 49 (46.7) |  |
| Severe | 27 (16.5) | 13 (22.0) | 14 (13.3) |  |
| Progression |  |  |  | 0.774 |
| Progress to moderate, n (%) | 20 (12.2) | 6 (10.2) | 14 (13.3) |  |
| Progress to severe, n (%) | 38 (23.2) | 15 (25.4) | 23 (21.9) |  |
| PaO2/FiO2 ratio, mmHg | 162.3 (111.8–221.5) | 134.4 (104.8–187.7) | 179.7 (129.4–237.0) | <0.001 |
| PaO2, mmHg | 127.7 (95.4–117.7) | 122.0 (87.1–171.7) | 129.4 (100.0–177.8) | 0.213 |
| FiO2, % | 100.0 (70.0–100.0) | 100.0 (87.5–100.0) | 100.0 (60.0–100.0) | 0.011 |
| pH | 7.39 (7.32–7.44) | 7.36 (7.28–7.43) | 7.39 (7.33–7.44) | 0.053 |
| PaCO2, mmHg | 37.9 (33.5–44.3) | 39.7 (35.7–46.8) | 36.8 (33.4–44.2) | 0.175 |
| V_T_, ml/kg PBW | 8.03 (7.08–8.88) | 7.58 (7.01–8.46) | 8.13 (7.14–9.31) | 0.023 |
| Minute ventilation, L/min | 9.0 (7.0–10.5) | 8.4 (6.8–9.9) | 9.1 (7.2–10.8) | 0.277 |
| PEEP, cmH2O | 8.0 (8.0–10.0) | 10.0 (8.0–12.0) | 8.0 (6.0–10.0) | <0.001 |
| Plateau pressure, cmH2O | 24.0 (20.0–27.0) | 26.0 (22.0–29.5) | 22.0 (18.5–26.0) | 0.002 |
| Driving pressure, cmH2O | 15.0 (12.0–18.0) | 14.0 (12.0–18.5) | 15.0 (11.0–18.0) | 0.892 |
| Static compliance, ml/cmH2O | 34.0 (25.5–42.2) | 32.0 (24.9–37.5) | 35.0 (25.9–43.0) | 0.104 |
| Mean airway pressure, cmH2O | 14.0 (11.0–16.0) | 14.8 (13.0–16.8) | 13.0 (11.0–16.0) | 0.001 |
| Ventilatory ratio | 1.57 (1.27–1.92) | 1.54 (1.27–1.92) | 1.66 (1.30–1.91) | 0.429 |
| Adjunctive therapy, n (%) |  |  |  |  |
| Prone positioning | 41 (25.0) | 33 (55.9) | 8 (7.6) | <0.001 |
| Recruitment maneuvers | 32 (19.5) | 22 (37.3) | 10 (9.5) | <0.001 |
| Inhaled Nitric Oxide | 13 (7.9) | 0 (0.0) | 13 (12.4) | 0.004 |
| ECMO | 3 (1.8) | 1 (1.7) | 2 (1.9) | 1.000 |

^e^ ARDS, acute respiratory distress syndrome; ^f^ PBW, predicted body weight; ^g^ PEEP, positive end-expiratory pressure; ^h^ ECMO, extracorporeal membrane oxygenation
